# Experimental Investigation on the Electrical Properties of DPPT-TT Polymer Field-Effect Transistors Featuring Stair Gate Dielectric

**DOI:** 10.3390/polym17030289

**Published:** 2025-01-23

**Authors:** Hong Zhu, Yi Qian, Yu Yan, Lijian Chen, Quanhua Chen, Huabin Sun, Yong Xu, Guangan Yang

**Affiliations:** College of Integrated Circuit Science and Engineering, Nanjing University of Posts and Telecommunications, Nanjing 210023, China; 2020020116@njupt.edu.cn (H.Z.); 1022223606@njupt.edu.cn (Y.Q.); max_njupt@163.com (Y.Y.); 2020020217@njupt.edu.cn (L.C.); 2021020307@njupt.edu.cn (Q.C.); hbsun@njupt.edu.cn (H.S.)

**Keywords:** PFETs, power devices, stair dielectric, breakdown voltage

## Abstract

P-type polymer field-effect transistors (PFETs) achieve wide applications due to their environmental compatibility and inherent flexibility. However, the dielectric in PFETs presents a vulnerability that restricts the development of the advancement of p-type power devices and power integrated circuits with high voltage in power devices. In this work, we provide a novel method that employs p-type polymer DPPT-TT high-voltage PFETs with a stair gate dielectric structure (SGD) at both the source and drain sides. The breakdown voltage of this device is significantly increased, rising from 19 V to 80 V. This improvement is attributable to the SGD structure’s ability to reduce the electric field between the source and drain. Although the step gate length (*L_SGD_*) is 50 μm, the on-state resistance only increases by 20% in comparison to conventional devices. The step region contributes an additional resistance of 2.5 × 10^4^ Ω/μm. The operational mechanism of the SGD PFET is demonstrated by TCAD simulations.

## 1. Introduction

Significant advancements in polymer field-effect transistors over recent decades have broadened their applications across diverse domains, including flexible sensors, active-matrix organic-light-emitting diodes, biological monitoring, and solar energy conversion [1,2,3,4,5]. These devices are highly valued for their substantial functionality, tunable properties, mechanical robustness, and reliable performance even under stringent environmental conditions [6,7,8,9,10]. Moreover, the simplicity of their fabrication, large-area uniformity, and cost-effectiveness have propelled the development of PFET-based integrated circuits, encompassing analog, digital, and radio frequency identification circuits, to address the variegated demands of everyday life [11,12,13,14,15]. Consequently, the technology of PFETs is instrumental in the progress of advanced electronics. However, the current shortfall in high-performing organic semiconductor-based power devices represents a key obstacle in the development of fully flexible electronic systems.

Nevertheless, to date, there are few reports on the polymer-based power devices. For the conventional bottom-gate structure PFETs, the breakdown usually occurs in the gate dielectric in the overlap region between the gate and drain, which is the weak point under a high-operation voltage for thin-film transistors. Thus, the breakdown voltage is determined by the thickness of the dielectric [16]. Employing a thick gate dielectric can raise the *BV*, but it will greatly increase the on-state resistance (*R_on_*) of the PFETs. An alternative approach involves implementing the drain-offset structure between the gate and the drain electrode to withstand the substantial voltage drop in high-voltage PFETs [17]. However, the offset region exhibits high resistance, leading to the degradation of the device’s *R_on_*. Silicon-based power device technology presents challenges in its application to PFETs, primarily due to the complexities associated with doping processes and the limitations in utilizing photolithography processes [18,19,20]. For the same reason, the fabrication of PFETs with complex structures is also challenging.

In this work, we fabricated bottom-gate top-contact PFETs with the SGD positioned on both the source and drain sides. The semiconductor material is used diketopyrrolopyrrole-based polymer (DPPT-TT). The electrical properties include breakdown voltage (*BV*), threshold voltage (*V*th), transconductance (*g*_m_), subthreshold swing (*SS*), and *R*_on_. The SGD structure effectively reduced the electric field between the gate and drain, leading to the increase of *BV*. The channel region outside the SGD maintained a layer-thin gate dielectric, maintaining excellent output current capabilities. Consequently, the SGD PFETs with an *L*_SGD_ of 50 μm achieved a *BV* of 84 V, which is more than four times that of conventional devices, while the *R*_on_ was 6.3 MΩ, only 1.3 times that of conventional devices. TCAD simulations were employed to provide an in-depth understanding of the operational mechanisms of SGD PFETs.

## 2. Experimental Section

**Figure 1:**
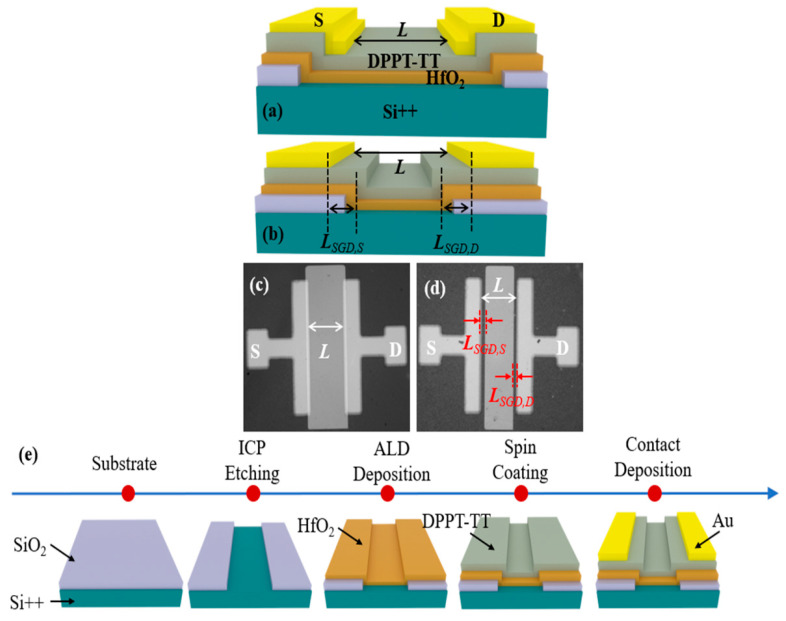
Schematic views of the (**a**) Conv. PFET and (**b**) SGD PFET. Optical images of the (**c**) Conv. PFET and (**d**) SGD PFET. (**e**) The fabrication process flow of the SGD PFETs.

A bottom-gate top-contact conventional polymer field-effect transistor (Conv. PFET) and the proposed 80-V SGD PFET are shown in Figure 1a,b, respectively. Figure 1c,d provides the optical images of the Conv. PFET and SGD PFET. The fabrication processes of SGD PFETs are shown in Figure 1e. The fabrication process began with a highly p-doped silicon wafer serving as the bottom gate, topped with a 50 nm thick layer of thermally grown silicon dioxide (SiO_2_) as the gate dielectric. The SiO_2_ was etched using an inductively coupled plasma etcher to create a trench. Subsequently, a 20 nm layer of hafnium dioxide (HfO_2_) was deposited via atomic layer deposition at 150 °C, forming the SGD structure combined with the SiO_2_. A 20 nm semiconductor layer of polymer DPPT-TT was then spin-coated onto the substrate and annealed at 150 °C for 1 h. Source–drain electrodes were formed by evaporating a 50 nm gold (Au) layer and patterning it with a hard mask. The SGD length (*L_SGD_*) is denoted as:

(1) $$L_{SGD}=L_{SGD,S}+L_{SGD,D}$$

where *L_SGD,S_* and *L_SGD,D_* are the stair length at the source and drain sides, respectively.

The SGD PFETs with *L_SGD_* of 50 μm (SGD-A), 100 μm (SGD-B), and 150 μm (SGD-C) were fabricated and investigated in this work. The key dimensions of the fabricated devices are shown in Table 1. All devices were fabricated with a channel length of 300 μm and a channel width of 1000 μm. The transfer and output characteristics of the PFETs were evaluated using a Keysight B1500 semiconductor precision analyzer. Essential parameters such as *V*th, *g*_m_, and *SS* were derived from the transfer curves. The drain breakdown voltages were individually monitored. The operational principles of the investigated device were elucidated through TCAD simulations.

## 3. Results and Discussion

**Figure 2:**
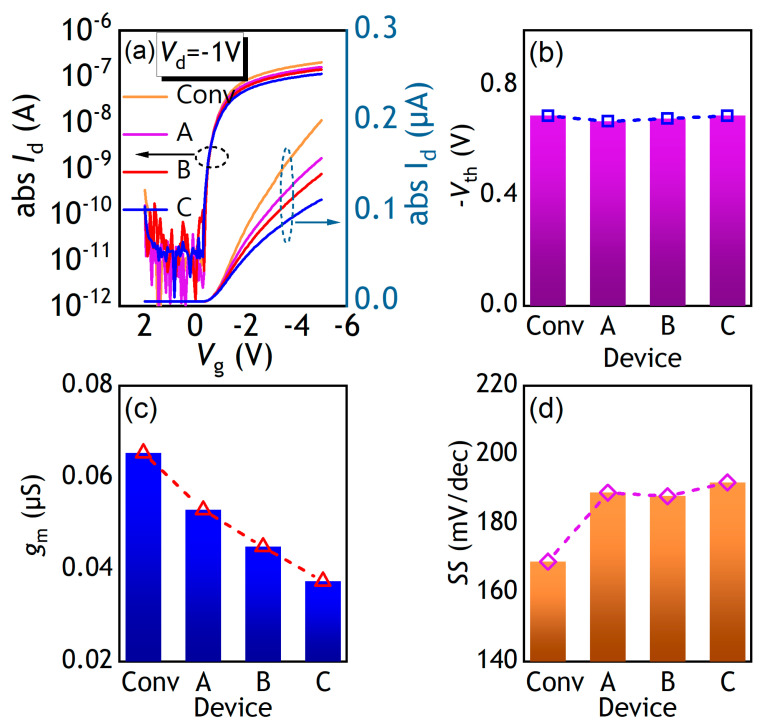
(**a**) Transfer curves of the conventional, SGD-A, SGD-B, and SGD-C PFETs. (**b**) *Vth*, (**c**) *g_m_*, and (**d**) *SS* versus the studied PFETs.

Figure 2a shows the transfer curves of the fabricated devices encompassing Conv., SGD-A, SGD-B, and SGD-C PFETs at the drain voltage (*V*_d_) of −1 V. The extracted *SS*, *V*th, and *g*_m_ of the PFETs are delineated in Figure 2b–d. All devices exhibit a threshold voltage of around −0.7 V in Figure 2b, there is a characteristic that can be related to the 20 nm thin dielectric layer, which enhances gate control. The *V*th of the Conv. PFETs and SGD PFETs with various *L_SGD_* are almost the same as each other. For the amorphous semiconductor-based channel material, the conduction threshold can be described as a transition of fermi level from deep to tail states [21]. The 50 nm SiO_2_ layer in the stair region increases the effective thickness of the gate dielectric (EOT), and EOT increases with the increasing *L*_SGD_. The weak reliance of the *V*th on EOT means the fabricated OSC film has a low density of deep states. In addition, the increase in EOT contributes to a reduced gate capacitance of the SGD PFETs. Thus, the *g*_m_ drops and decreases with the *L*_GSD_, as depicted in Figure 2c. Correspondingly, a little increase in *SS* for the SGD PFETs was caused by the decreasing gate capacitance, as shown in Figure 2d.

**Figure 3:**
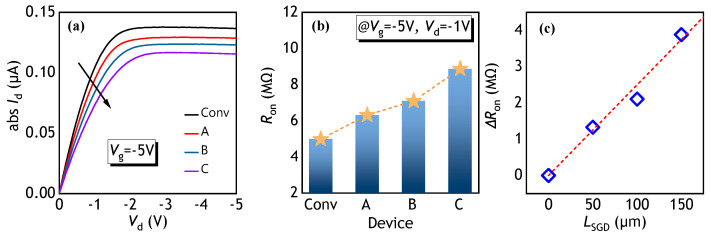
(**a**) Output curves of the conventional, SGD-A, SGD-B, and SGD-C PFETs at the *V_g_* of −5 V. (**b**) Extracted *R_on_* in the linear region and (**c**) Δ*R*_on_ versus *L_GSD_* for the studied PFETs.

Figure 3a shows the output curves at the gate voltage (*V*_g_) of −5 V for the Conv., SGD-A, SGD-B, and SGD-C PFETs. There is no current crowding at a low *V*_d_ for these PFETs. The decrease in *I_d_* for the increasing *L*_SGD_ is noted. In the stair region of the SGD PFET, the gate dielectric thickness surpasses that of the Conv. PFET, resulting in a low electron concentration induced by the same *V*_g_. The drop in carrier density elevates the resistance of the channel, intensifying with increasing *L_SGD_*, consequently diminishing the output current. Figure 3b illustrates *R*_on_ at the *V*_d_ of −1 V and *V*_g_ of −5 V in the linear region extracted from the transfer curves. When the *L*_SGD_ increases, the *R*_on_ level exhibits an increasing trend. For SGD-A PFET with an *L*_SGD_ of 50 μm, the *R*_on_ measures 6.3 MΩ, only 1.3 times the *R*_on_ of 5.0 MΩ of the Conv. PFET. The channel area outside the SGD region in the SGD-A PFET comprises a 20 nm thick HfO_2_ dielectric, maintaining a high concentration of the accumulated carriers and sustaining a low *R*_on_. Figure 3c shows the Δ*R*_on_ as the function of *L*_GSD_, extracted from Figure 3b. Extracting the on-state resistance from Figure 3b and plotting the change in on-state resistance separately, it can be observed that the increase in conduction resistance caused by the SGD structure exhibits a nearly linear relationship of a slope of 2.5 × 10^4^ Ω/μm. This indicates that the SGD structure does not have a particularly significant impact on the on-state resistance.

**Figure 4:**
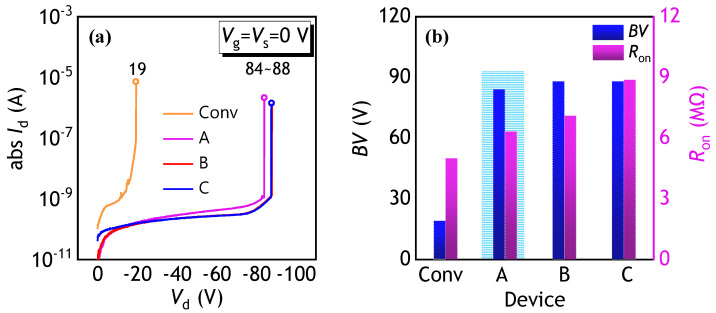
(**a**) *I_d_* versus the *V_d_* for the conventional, SGD-A, SGD-B, and SGD-C PFETs. The breakdown was measured by sweeping the *V_d_* with the *V_g_* and vs. grounded. (**b**) The obtained *BV* and *R_on_* of the PFETs.

The breakdown characteristics of the proposed PFETs are examined. The *I*_d_-*V*_d_ curves at both the *V*_g_ and the source voltage of 0 V are presented in Figure 4a. The breakdown occurs in a hard breakdown mode, which is irreversible. Furthermore, the extracted *BV* and *R*_on_ of the studied PFETs are depicted in Figure 4b. Conv. PFETs, with only a 20 nm thick HfO_2_ between the gate and drain, exhibit the lowest breakdown voltage of 19 V. In contrast, PFETs with the SGD structure, which combines a 50nm SiO_2_ layer with a 20 nm HfO_2_ in the overlap region, see a significant increase in breakdown voltage. In an ideal situation, the electric displacement vector is continuous at the interface between different dielectric layers. When a drain voltage is applied, the electric field in the dielectric layer with a higher dielectric constant (HfO_2_) is lower than that in the dielectric layer with a low dielectric constant (SiO_2_). When the thickness of SiO_2_ is higher than that of HfO_2_, most electric field is endured by the SiO_2_, leading to a higher breakdown voltage. All SGD PFETs achieve similar *BV* values due to the constant SGD thickness in the stair region of these devices. The SGD-A PFET stands out with a *R*_on_ of 6.3 MΩ and a *BV* of 84 V, exhibiting superior HV performance compared to its counterparts.

The experimental findings indicate that the SGD PFET can achieve a favorable trade-off between *R*_on_ and *BV*. To gain deeper insights into the operational mechanisms of the SGD PFETs, simulations using TCAD_Silvaco were undertaken. Table 2 summarizes the material simulation parameters for the OSC-based semiconductor, encompassing the energy band gap, electron affinity, effective density of states in the highest occupied molecular orbital (HOMO) and lowest unoccupied molecular orbital (LUMO), as well as the gate dielectric permittivity and contact work function.

The subgap density of states (DOS) model, comprising four parameterized components—namely, the acceptor-like exponential function, the acceptor-like Gaussian function, the donor-like exponential function, and the donor-like Gaussian function—proves instrumental in characterizing trap DOS within the bulk OSC-based channel material. In the case of OSC materials, exponential DOS is applied to represent tail states close to the conduction band edge and valence band edge, whereas Gaussian DOS functions are employed to characterize deep gap states.

Considering that the PFETs operate in p-type mode, we considered mainly the donor-like states in simulation. The donor-like exponential DOS is depicted by [22]:

(2) $$g_{TD}\left(E\right)=N_{TD}\exp\left(\frac{E_{HOMO}-E}{W_{TD}}\right)$$

where *E_HOMO_* is the energy level of HOMO and *E* is the energy level. *N_TD_* is the donor-type intercept DOS at *E*_HOMO_, and *W*_TD_ is the characteristic decay energy. The donor-like Gaussian DOS is given by:

(3) $$g_{GD}\left(E\right)=N_{GD}exp\left[-{\left(\frac{E-E_{GD}}{W_{GD}}\right)}^{2}\right]$$

where *N*_GD_ is the trap density at the central energy *E*_GD_ of the Gaussian distribution, and *W*_GD_ is the characteristic decay energy. The essential fitting parameters of the DOS model are detailed in Table 3.

**Figure 5:**
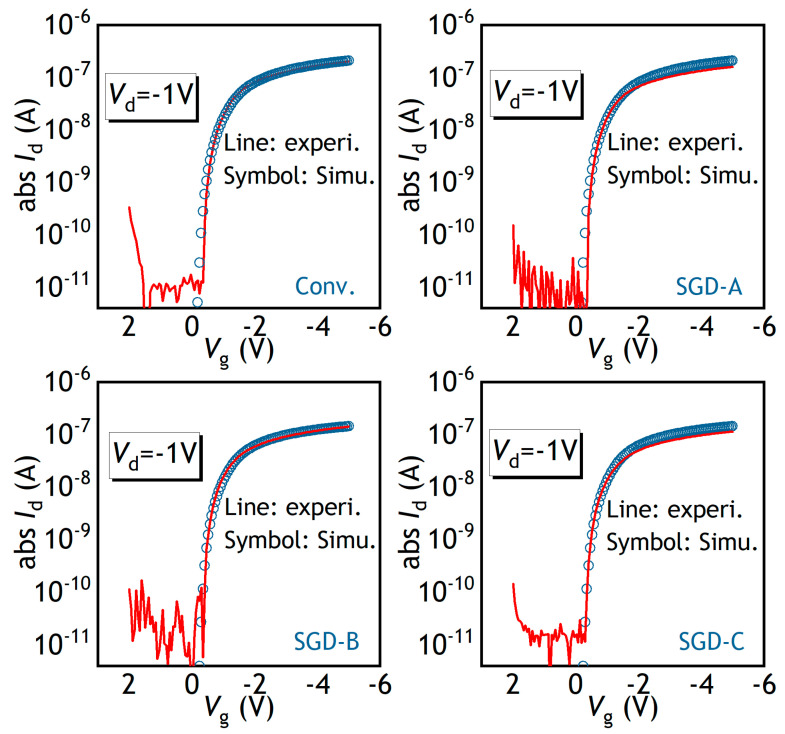
TCAD simulation fitting results for the transfer curves of conventional, SGD-A, SGD-B, and SGD-C PFETs at *V*_d_ of −1 V, respectively.

Figure 5 shows the comparison between the transfer characteristics of experimental data and simulated results about SGD-A, SGD-B, and SGD-C PFETs. The simulations exhibit a satisfactory alignment with the experimental data.

**Figure 6:**
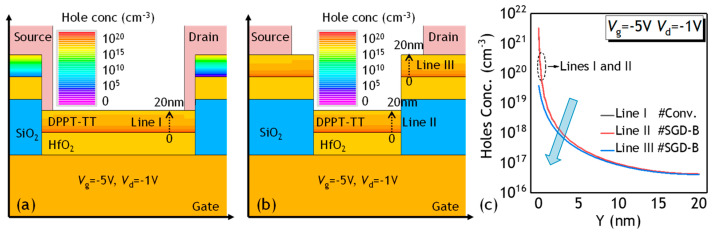
Simulated results on the hole concentration distribution of (**a**) Conv. PFET, and (**b**) SGD-B PFET with the *L_SGD_* of 100 μm. (**c**) Hole concentration along lines I-III.

Figure 6 depicts the simulated distribution of hole concentration in the DPPT-TT material within both the Conv. PFET and SGD-B PFET at the *V*_g_ of −5 V and *V*_d_ of −1 V. The hole concentration profiles along lines I in the channel of the Conv. PFET, as well as along lines II and III in the channel of the SGD-B PFET, are presented in Figure 6c. For the SGD-B PFET, the carrier concentration along line III appears lower than that along line II, indicating reduced channel carrier density in the stair region compared to regions outside the stair region. The diminished capacitance of the thick SGD featuring a bilayer gate dielectric results in decreased gate-induced carriers within the stair channel region, thereby elevating channel resistance. Consequently, the output current of the SGD PFET falls below that of the Conv. PFET. It is significant to observe that lines I and II exhibit identical carrier distributions. Specifically, the gate dielectric beneath the active layer beyond the stair region consists of a 20 nm HfO_2_ layer, mirroring the setup in the Conv. PFET. Under the same *V*_g_, the induced holes in the channel region with the singular dielectric are on the same level as that of Conv. PFET. The channel outside the stair region with a high carrier concentration, ensures the low sacrifice in current for the power SGD PFET.

**Figure 7:**
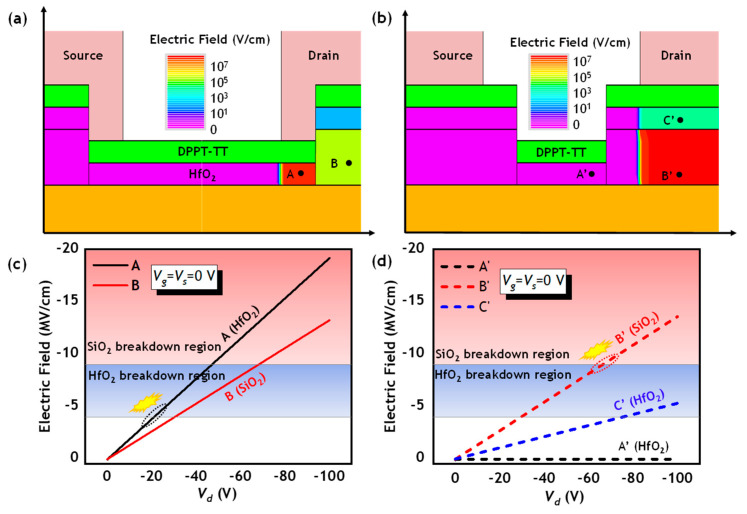
Simulated results on the electric field distribution in the dielectric of (**a**) Conv. PFET, and (**b**) SGD-B PFET with the *L_SGD_* of 100 μm at the *V*_g_ = *V*_s_ = 0 V, *V*_d_ = −100 V. The electric field at randomly selected points in the gate and drain overlap region. Electric field versus *V*_d_ at positions (**c**) A, B in Conv. PFET, (**d**) A’ B’, and C’ in SGB-B PFET. The blue area is the breakdown region of HfO_2_, and the red area is the breakdown region of SiO_2_.

Figure 7a,b show the simulation results illustrating the distribution of electric fields at the *V*_g_ = *V*_s_ = 0 V and *V*_d_ = 100 V within the dielectric for the Conv. PFET and SGD-B PFET, respectively. The electric field, varying with *V*_d_ at positions A and B in the Conv. PFET and A’, B’, and C’ in SGD-B PFET, are shown in Figure 7c,d correspondingly. In the Conv. PFET, the electric field at position A within the HfO_2_ surpasses that at position B within the SiO_2_ and reaches a peak of 4 MV/cm at the *V*_d_ of −20 V, nearing the critical electric field of HfO_2_, resulting in a limited *BV*. Conversely, in the SGD-B PFET, the SGD structure diminishes the electric field at position A’ within the HfO_2_, identified as a vulnerable point in Conv. PFETs when operating at high *V*_d_, by optimizing the distribution of electric fields. Positions A’ and C’ experience subdued electric fields at elevated *V*_d_ levels. The electric field at position B’ in the stair region, featuring a thicker total gate dielectric, absorbs the majority of the electric field at higher *V*_d_, thereby elevating the *BV*.

The developed SGD PFETs significantly raise the *BV* and can reduce *R*_on_ by reducing the *L*_SGD_, showcasing the substantial potential for application in power management circuits.

## 4. Conclusions

In this study, we introduce a novel approach involving p-type high-voltage PFETs that incorporate an SGD structure at both the source and drain terminals. The polymer DPPT-TT was deposited via spin-coating onto the SGD consisting of HfO_2_ and SiO_2_ layers to fabricate power SGD PFETs. Notably, the *BV* of the proposed device was elevated from 19 V to over 80 V. The SGD structure effectively reduces the electric field between the drain and gate, thereby enhancing the *BV*. With an SGD length of 50 μm, the *R*_on_ only experienced a modest 20% decline compared to conventional devices. Through TCAD simulation, the operational principles of the SGD PFETs were elucidated. This approach allows for the simultaneous achievement of high *BV* and low *R*_on_ in power PFETs.

## Figures and Tables

**Table 1:** Key dimensions of the fabricated devices.

Device	Conv	A	B	C
*t_HfO__2_* (nm)	20	20	20	20
*t_SiO__2_* (nm)	/	50	50	50
*L_SGD_* (μm)	/	50	100	150

**Table 2:** Key simulation parameters of the material.

Energy band gap (eV)	1.26
Electron affinity (eV)	4.07
Effective density of states (HOMO) (cm^−3^)	1.0 × 10^21^
Effective density of states (LUMO) (cm^−3^)	1.0 × 10^21^
Permittivity of HfO_2_	21
Permittivity of SiO_2_	3.9
Work function of contacts (eV)	5.1

**Table 3:** Key simulation parameters of the bulk defects model.

N_TD_ (cm^−3^eV^−1^)	W_TD_ (eV)	N_GD_ (cm^−3^eV^−1^)	W_GD_ (eV)	E_GD_ (eV)
1.0 × 10^18^	0.1	1.0 × 10^16^	0.1	0.4

## Data Availability

The original contributions presented in this study are included in the article. Further inquiries can be directed to the corresponding authors.
